# A Cryopreservation and Regeneration Protocol for Embryogenic Callus of *Larix olgensis*

**DOI:** 10.3390/plants14203127

**Published:** 2025-10-10

**Authors:** Chen Wang, Wenna Zhao, Yu Liu, Hao Dong, Yajing Ning, Chengpeng Cui, Hanguo Zhang, Meng Li, Shujuan Li

**Affiliations:** State Key Laboratory of Tree Genetics and Breeding, Northeast Forestry University, Harbin 150040, China

**Keywords:** preculture, cryoprotectant, thawing temperature, cell viability, proliferation rate, germplasm preservation

## Abstract

*Larix olgensis* is a valuable timber species in northern China, typically propagated through somatic embryogenesis (SE). However, long-term subculture can lead to a loss of embryogenic potential. This study aimed to establish a simple and stable protocol for the cryopreservation and regeneration of *L. olgensis* embryogenic callus (EC) that preserves its SE potential and regenerative capacity. The slow-freezing method was employed for cryopreservation. A cryopreservation protocol for *L. olgensis* EC was developed by optimizing the preculture duration and conditions, cryoprotectant composition and thawing temperature. The results showed that optimal outcomes were achieved using a 24 h stepwise preculture on medium containing 0.2 and 0.4 mol∙L^−1^ sucrose, followed by cryoprotectant treatment with 0.4 mol∙L^−1^ sucrose, 2.5% (*v*/*v*) dimethyl sulfoxide (DMSO) and 10% polyethylene glycol 6000 (PEG_6000_), and thawing at 37 °C. EC cryopreserved using this protocol achieved a 100% recovery rate. Moreover, the cryopreserved recoverable EC successfully underwent SE, progressing through germination and rooting. Cryopreservation duration (storage duration in liquid nitrogen) did not affect cell viability and proliferation rate, confirming the protocol’s suitability for long-term cryopreservation of *L. olgensis* EC. This study provides a valuable reference for the cryopreservation and regeneration of *L. olgensis* EC, with potential applications for other coniferous species. It establishes a robust foundation for the large-scale propagation of conifers.

## 1. Introduction

*Larix olgensis* is a commercially valuable timber species in northern China and also possesses significant ecological importance. It contributes to soil and water conservation, air purification, and noise reduction and provides habitats for wildlife, playing an irreplaceable role in maintaining ecological balance [1]. Somatic embryogenesis (SE) is a process in which structures similar to zygotic embryos are formed directly from somatic cells without gamete fusion [2]. Compared to conventional breeding methods, SE offers several advantages, including high propagation efficiency, enhanced genetic stability, shorter breeding cycles, and lower production costs [3]. Furthermore, SE is highly controllable and easy to observe, making it valuable for both theoretical and applied research [4]. To date, SE has been successfully applied in *L. olgensis* for rapid propagation, germplasm preservation, and genetic improvement [5]. However, the long-term subculture of embryogenic callus (EC) leads to a series of adverse effects on subsequent development. These effects manifest as reduced cell viability, loss of regenerative capacity, alterations in gene expression patterns, and morphological and physiological abnormalities in regenerated plantlets [6]. These issues significantly impair the efficiency and stability of tissue regeneration. Cryopreservation refers to the long-term preservation of plant organs, tissues, or cells at the ultra-low temperature of −196 °C (typically in liquid nitrogen) [7]. At this temperature, EC are in metabolic standstill or suspended animation, in the case of the kinetic energy, enzymatic reactions, and molecular motion within biological systems, diminishing or ceasing entirely [8]. This technique is commonly integrated with in vitro culture methods. Therefore, the development of successful micropropagation systems is critical for cryopreserving propagules [9,10]. This enables the long-term, secure preservation of diverse materials, including pollens, calli, somatic embryos, zygotic embryos, seeds, shoot tips, and dormant buds [11,12,13,14]. Consequently, cryopreservation is universally recognized as the most effective and ideal method for the long-term preservation of plant germplasm resources [15]. The integration of SE and cryopreservation technologies shortens forest tree breeding cycles by allowing long-term cryopreservation of EC from diverse genotypes in liquid nitrogen. This approach minimizes the risks of somaclonal variation and reduces the labor and resources required for repeated subculturing [16]. Concurrently, elite genotypes identified through clonal field trials can be retrieved from cryopreservation, thawed, and mass-propagated via SE. This enables the rapid deployment of somatic embryo-derived plantlets in clonal forestry operations [17].

Since its initial development, cryopreservation protocols have been continuously refined. Current methods can be broadly classified into three categories: conventional techniques such as rapid-freezing [18] and slow-freezing [19] (used for over 40 years); vitrification-based approaches, including droplet vitrification [20], encapsulation–vitrification [21], and standard vitrification [22] and alternative methods such as air desiccation-freezing [23] and cryoplate protocols [24,25]. Each method has distinct advantages and limitations, and their suitability varies across plant species. Among these, conventional slow-freezing and vitrification-based methods are the most widely used in plant cryopreservation, with slow-freezing being particularly common in coniferous species [26,27,28,29,30]. The cryopreservation and regeneration process involves multiple stressors, including osmotic stress, chemical toxicity, ice crystallization, and cold shock, which collectively threaten cellular integrity and compromise preservation efficacy [24]. To mitigate these challenges, the three critical steps of preculture, cryoprotection, and optimized thawing form the foundation of successful cryopreservation. Preculture enables controlled cellular dehydration, reducing intracellular water content to enhance tolerance against rapid temperature fluctuations and extreme desiccation [27]. Cryoprotectants further improve preservation efficiency through synergistic mechanisms that establish a biological protection barrier during dehydration and low-temperature stress, primarily by inhibiting lethal ice crystal formation and maintaining membrane integrity [31]. Additionally, tailored thawing protocols prevent destructive ice recrystallization during rewarming, ensuring structural and functional recovery of preserved tissues [24]. Cryopreservation serves as a critical technique for the long-term and efficient conservation of forest tree germplasm resources. Its efficiency is co-regulated by multiple factors, including explant type, genotype, tissue culture procedure, and cryopreservation methodology [32]. Therefore, species-specific protocol optimization is essential to improve cryopreservation outcomes. Although *L. olgensis* is an important coniferous species in northern China, cryopreservation techniques for its EC have not yet been established. Our laboratory has successfully developed a stable SE system for *L. olgensis* [33], providing a consistent material basis for such studies. Therefore, the preservation of elite embryogenic cell lines is urgently required.

This study aims to establish a simple and efficient cryopreservation protocol by optimizing key steps in the cryopreservation process (including preculture, cryoprotectant, and thawing temperature). The goal is to maximize the retention of somatic embryogenic potential, thereby laying a crucial foundation for the long-term cryopreservation of elite germplasm resources in *L. olgensis*.

## 2. Results

### 2.1. Optimization of 2,3,5-Triphenyltetrazolium Chloride (TTC) Concentration for Cell Viability Assay

**Figure 1:**
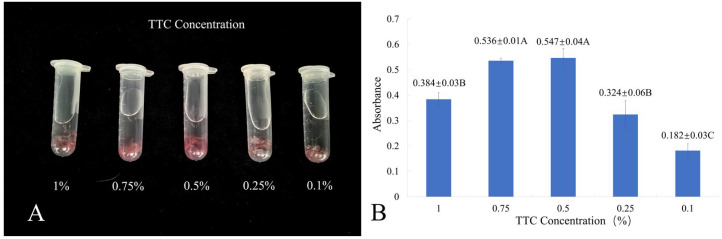
Absorbance and coloration comparison of *L. olgensis* EC under different TTC concentrations. (**A**) coloration comparison; (**B**) absorbance. Different uppercase letters indicate significant differences at the *p* < 0.01 level.

TTC is reduced by hydrogen in biological materials to form the insoluble red compound triphenylformazan. EC incubated in varying TTC concentrations under dark conditions for 24 h exhibited differential red coloration intensity (Figure 1A). The 0.5% TTC treatment yielded the deepest coloration, contrasting with the lightest color at 0.1%.

The TTC reduction assay is a widely used method for assessing cell viability [34]. The results showed highly significant differences in absorbance among the TTC concentrations tested (Figure 1B). The highest absorbance value was observed at 0.5% TTC (0.547), followed by 0.75% TTC (0.536), and the lowest at 0.1% TTC (0.182). These quantitative findings are consistent with the qualitative color observations.

### 2.2. Screening of Optimal Preculture and Cryoprotectant Protocols

#### 2.2.1. Effects of Preculture and Cryoprotectant Protocols on Cell Viability

Range analysis (R-values, Table 1) revealed that the type of preculture medium had the greatest effect on the viability of *L. olgensis* EC (R = 1.931%), followed by preculture duration (R = 1.569%) and dimethyl sulfoxide (DMSO) concentration (R = 1.389%), while polyethylene glycol 6000 (PEG_6000_) concentration had minimal impact (R = 0.458%). Among all treatments, treatment 12 (a 24 h stepwise preculture on medium containing 0.2 and 0.4 mol∙L^−1^ sucrose, followed by cryoprotectant treatment with 0.4 mol∙L^−1^ sucrose, 5% (*v*/*v*) DMSO, and 10% PEG_6000_) resulted in significantly higher cell viability than the others.

Multiple comparisons (Table 2) showed that the highest viability (3.486%) was achieved with a 24 h preculture, which was significantly higher than the lowest viability (1.917%) observed at 12 h. No significant differences were found between 24 h and 36 h, or between 12 h and 48 h, but the difference between 24 h and 12 h was highly significant. Stepwise preculture on sucrose-containing medium resulted in a viability of 3.875%, which differed highly significantly from all other treatments. The highest viability under DMSO treatment (3.569%) was obtained at 2.5% concentration. This result was not significantly different from that of the 5% DMSO group, but was significantly lower than those treated with 10% and 15% DMSO. For PEG_6000_, the maximum viability (3.111%) occurred at 10% concentration, with no significant differences across the concentration gradient.

In summary, the optimal cryopreservation protocol for *L. olgensis* EC entails that the EC undergo 24 h preculture on a medium supplemented with 0.2 mol∙L^−1^ of sucrose, followed by a medium supplemented with 0.4 mol·L^−1^ sucrose. Subsequently, they are immersed in cryoprotectant solution containing 0.4 mol·L^−1^ sucrose, 2.5% DMSO, and 10% PEG_6000_. Since this specific combination was not included in the original 16 treatments of the orthogonal array testing strategy (OATS), further experimental validation was conducted to confirm its effectiveness in minimizing cryoinjury.

Under experimental conditions identical to the 16 OATS treatments, the same weight of EC (0.6 g) was cryopreserved using the optimized protocol. Cell viability was assessed with three replicates. Results demonstrated that EC preserved with the optimized protocol showed a post-thaw viability of 5.111% (Table 3). In contrast, the directly cryopreserved control showed only 1.111% viability, indicating a highly significant difference between the two groups.

#### 2.2.2. Effects of Preculture and Cryoprotectant Protocols on Proliferation Rate

The proliferation rate of post-thaw EC reflects its recovery efficacy. This study quantified proliferation rates after 4 weeks of recovery for both the 16 OATS treatments and the optimized protocol (Table 4). The control exhibited no proliferation, whereas the optimized protocol achieved the highest proliferation rate (710%), followed by treatment 12 (480%). These results demonstrate that the optimized protocol minimizes cryoinjury and maximizes post-cryopreservation recovery.

### 2.3. Screening of Optimal Thawing Temperature

**Figure 2:**
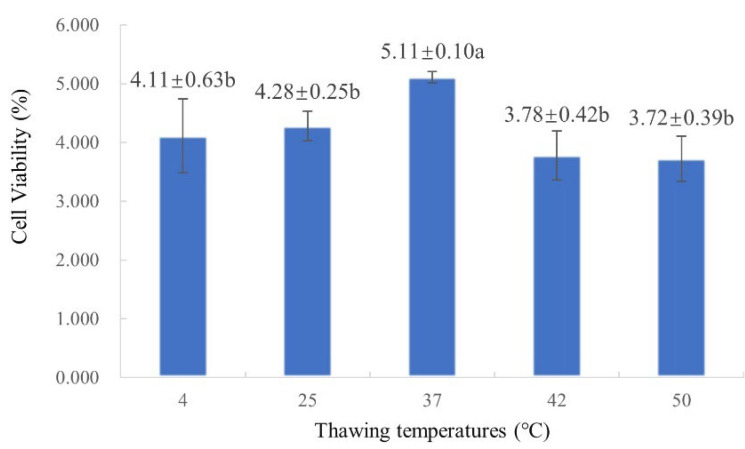
Cell viability of *L. olgensis* EC under different thawing temperatures. Different lowercase letters indicate significant differences at the *p* < 0.05 level.

As shown in Figure 2, cell viability initially increased with rising thawing temperature, reaching a maximum of 5.11% at 37 °C, and then decreased at higher temperatures. The cell viability at 37 °C showed significant differences from that at all other temperatures tested. Therefore, 37 °C was selected as the optimal thawing temperature in this protocol.

### 2.4. Recovery Rate Assessment

**Figure 3:**
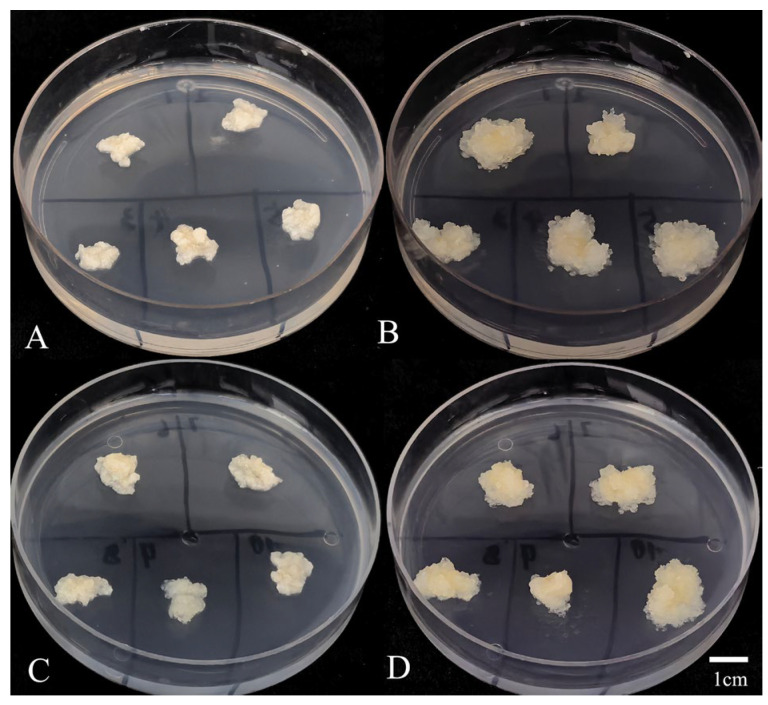
*L. olgensis* EC recovery 4 weeks post-thaw. (**A**,**C**) EC after thaw; (**B**,**D**) EC recovery for 4 weeks.

Ten clumps of EC cryopreserved for 4 months using the optimized protocol (a 24 h stepwise preculture on medium containing 0.2 and 0.4 mol∙L^−1^ sucrose, followed by cryoprotectant treatment with 0.4 mol∙L^−1^ sucrose, 2.5% DMSO and 10% PEG_6000_) were thawed at 37 °C, and initial weights were recorded (Figure 3A,C). After recovery of 10 days, translucent white EC emerged. Following 4 weeks of recovery, all 10 clumps showed noticeable weight gain and were covered with a layer of translucent white callus (Figure 3B,D), confirming a 100% recovery rate. The emergence of new proliferative tissues confirms that the cryopreserved EC has successfully resumed normal growth under the optimized protocol.

### 2.5. Effect of Cryopreservation Duration on Cell Viability and Proliferation

**Figure 4:**
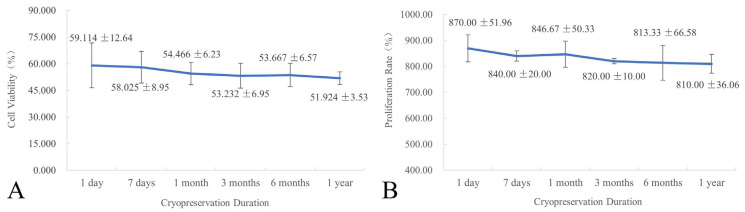
Effect of cryopreservation duration on cell viability or on the proliferation rate of *L. olgensis* EC. (**A**) cell viability; (**B**) proliferation rate.

To validate the long-term stability of this protocol for *L. olgensis* EC, samples cryopreserved for 1 day, 7 days, 1 month, 3 months, 6 months, and 1 year were thawed and assessed for cell viability (Figure 4A) and proliferation rate (Figure 4B). The results showed no significant differences in either cell viability or proliferation rate across the different cryopreservation durations, indicating negligible impact on post-thaw regenerative growth of EC. This protocol effectively minimized cryoinjury during extended cryopreservation, achieving stable long-term preservation of plant genetic resources.

### 2.6. SE and Germination After Cryopreservation

**Figure 5:**
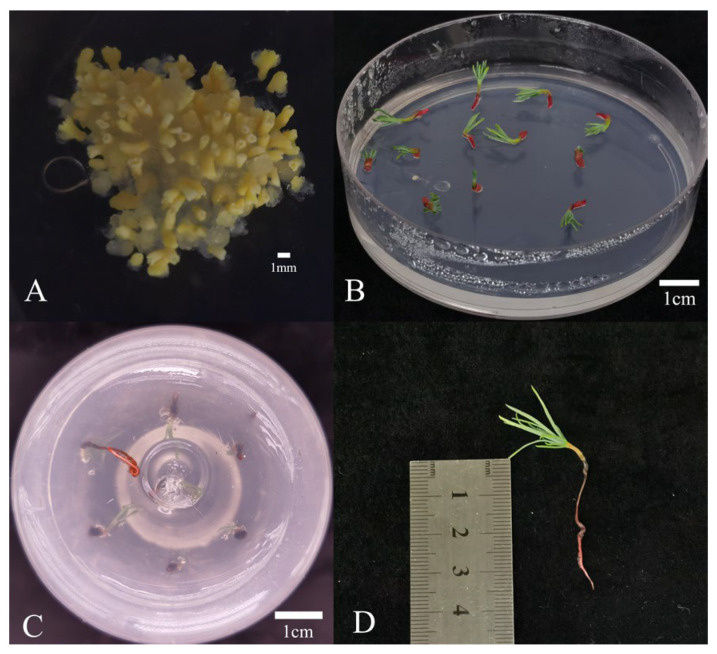
SE from cryopreserved-regenerated EC of *L. olgensis*. (**A**) somatic embryos maturation (40 days); (**B**) somatic embryo germination (5 days); (**C**) root elongation (20 days); (**D**) plantlet formation.

To evaluate the embryogenic potential after cryopreservation, EC that resumed normal proliferation was used in SE, germination, and plant regeneration assays. The cryopreserved EC retained the ability to undergo normal SE (Figure 5A). Crucially, somatic embryos derived from cryopreserved-recovered EC displayed normal morphology and developed into viable plantlets (Figure 5B–D). These results demonstrate that cryopreservation not only preserves proliferative competence but also maintains full embryogenic differentiation potential.

### 2.7. Flowchart of the Optimized Protocol

**Figure 6:**
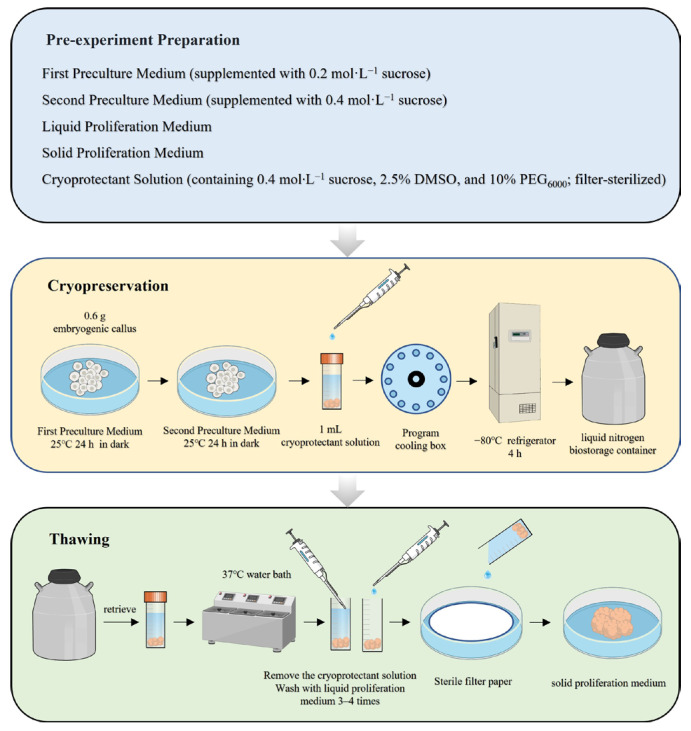
Cryopreservation procedure for EC of *L. olgensis*.

Based on the experimental results described above, a flowchart detailing the optimized cryopreservation procedure for *L. olgensis* EC is presented in Figure 6.

## 3. Discussion

This study successfully established a simple, efficient, and reliable protocol for the cryopreservation and regeneration of *L. olgensis* EC, addressing a critical gap in the preservation of this economically and ecologically important conifer.

A particularly notable result was the 100% recovery rate observed across all EC samples treated with the optimized protocol. Morphological evaluation showed complete encapsulation by translucent white callus after four weeks of recovery, highlighting the importance of integrating multiple protective strategies. The stepwise preculture using a sucrose gradient (0.2 mol∙L^−1^ followed by 0.4 mol∙L^−1^) under dark conditions at 25 °C promoted controlled cellular dehydration, which is essential for reducing osmotic shock and preventing intracellular ice formation during cryopreservation. This principle is supported by studies in other species, such as *Castanea dentata* [35], *Castanea sativa* [36], *Robinia pseudoacacia* [37], and *Vitis* spp. [38]. This was further enhanced by the composite cryoprotectant solution (0.4 mol∙L^−1^ sucrose, 2.5% DMSO, and 10% PEG_6000_). The combination of permeating (DMSO) and non-permeating (sucrose, PEG) cryoprotectants is consistent with established cryobiological theory [39]. *Larix gmelinii* var. [27] treated with this strategy (0.4 mol∙L^−1^ sorbitol + 5% DMSO) achieved a 70% regeneration rate. While the permeable agent DMSO is indispensable, its concentration must be carefully optimized to balance effectiveness and toxicity. In the present study, a lower concentration (2.5%) helped minimize DMSO cytotoxic effects, a concern also reported in *Taxodium hybrid* ‘zhongshanshan’ [40]. In the EC cell suspension of *Taxodium hybrid* ‘zhongshanshan’, DMSO concentrations were tested at 0%, 2.5%, 5%, 7.5%, 10%, and 12.5%. The results demonstrated that DMSO concentration significantly influenced both recovery time and callus proliferation. The most favorable recovery effect was observed with 10% DMSO following 60 min incubation on ice, which led to the shortest recovery period (5 days) and a high callus proliferation rate of 4.30. The inclusion of PEG is thought to enhance extracellular vitrification and stabilize membranes, further protecting cellular integrity [41]. Furthermore, rapid thawing in a 37 °C water bath resulted in the highest post-thaw viability (5.11%). This result is consistent with the fundamental cryobiological principle that rapid warming minimizes destructive ice recrystallization [42]. This aligns with findings in *Olea europaea* [43], and underscores the need for species-specific optimization at this step [12,27]. The highly significant difference in viability between the optimized protocol (5.11%) and direct cryopreservation controls (1.111%) clearly demonstrates the effectiveness of our protocol in reducing cryoinjury. While high TTC-reduction activity indicates viable cells with active metabolism, its discrepancy with proliferation capacity (e.g., in Treatment 1) highlights that only protocols that also preserve division competence, such as our optimized protocol, enable true long-term regeneration. A pivotal advantage of our protocol is its capability for long-term preservation without loss of biological quality. The absence of significant differences in cell viability and proliferation rate across cryopreservation durations from 1 day to 1 year confirms that cellular viability and proliferative capacity are stably maintained in liquid nitrogen. This stability is a cornerstone of successful cryopreservation [12]. It effectively eliminates the risks of somaclonal variation and loss of embryogenic competence inherent to chronic subculturing [6], thereby enabling the establishment of a reliable cryobank for *L. olgensis* genetic resources. Perhaps the most compelling evidence for the preservation of complete cellular function and totipotency is the ability of the regenerated EC to undergo normal SE. This ability confirms that the protocol not only preserves viability but also retains the differentiation capacity, which is essential for large-scale clonal propagation programs. The ability of the recovered EC to undergo SE is especially significant, given the previous work on *L. olgensis* that established methods for EC induction and SE [33], and Agrobacterium-mediated transformation [5].

In conclusion, this study develops the first comprehensive, highly efficient cryopreservation protocol for *L. olgensis* EC. Its success is evidenced by complete regeneration, stable long-term preservation, and maintained embryogenic potential. This protocol provides a powerful tool for the long-term conservation of elite genotypes of this important species. This supports advanced breeding programs, safeguards genetic diversity, and facilitates the deployment of clonal forestry. Future research could focus on employing techniques such as flow cytometry or SSR markers to conduct molecular verification of the genetic stability in *L. olgensis* following cryopreservation, and applying this validated protocol to a wider range of coniferous species.

## 4. Materials and Methods

### 4.1. Plant Material

The EC induced and subcultured for 6 months in our laboratory served as the experimental material [33,44]. The fresh EC selected and stabilized on proliferation medium for 3–5 days was used for subsequent experiments.

### 4.2. Culture Medium Formulation

All culture media (Table 5) were formulated using BM (Basal Medium) and MS (Murashige and Skoog) as the basal media [45].

### 4.3. Methods

#### 4.3.1. Optimization of TTC Concentration

Cell viability refers to the percentage of living cells within a cell population, reflecting the overall health status of the cells [46]. Cell viability served as the key metric for determining the optimal cryopreservation protocol for EC [27].

Cell viability of *L. olgensis* EC was assessed using the TTC reduction assay, which primarily reflects dehydrogenase activity in living cells. The viability is estimated based on the intensity of red color formation resulting from the reduction of TTC to formation [47]. Fresh EC (0.1 g), not subjected to cryopreservation, was placed into 5 mL centrifuge tubes. Then, 1.5 mL of TTC solutions at concentrations of 0.1%, 0.25%, 0.5%, 0.75%, and 1% were added. The tubes were incubated in the dark at 25 °C for 24 h. Three independent replicates were performed for each concentration. After incubation, the staining intensity was visually assessed. The TTC solution was discarded, and any residual solution was removed by rinsing the EC with distilled water. EC was incubated in 3 mL of 95% ethanol at 65 °C for 30 min, and the absorbance of the resulting supernatant was then measured at 485 nm.

#### 4.3.2. Preculture and Cryoprotectant Treatment

A total of 0.6 g EC was spread evenly onto a preculture medium for either stepwise preculture (first preculture on 0.2 mol∙L^−1^ sucrose or sorbitol, second preculture on 0.4 mol∙L^−1^ sucrose or sorbitol) or non-stepwise preculture (on 0.4 mol∙L^−1^ sucrose or sorbitol). All cultures were maintained in the dark at 25 °C for 12 h, 24 h, 36 h, or 48 h (Table 6). After preculture, the EC from different treatment groups were transferred into 2 mL cryovials, and 1 mL of filter-sterilized cryoprotectant solution was added at 25 °C. The cryoprotectant solution was formulated with DMSO (2.5%, 5%, 10%, 15%) and PEG_6000_ (0%, 5%, 10%, 15%) as base protectants, supplemented with 0.4 mol∙L^−1^ sucrose or sorbitol. The cryovials containing the cryoprotectant were then immediately transferred into a program cooling box (biosharp BS-02-CFC). The program cooling box commenced at a cooling rate of −1 °C∙min^−1^ until reaching −80 °C. After maintaining at −80 °C for at least 4 h, the cryovials were removed and rapidly plunged into a liquid nitrogen biological container for cryopreservation. Four factors, preculture duration, preculture strategy, DMSO concentration in the cryoprotective solution, and PEG_6000_ concentration, were evaluated, each at four levels. Sixteen treatment combinations were generated using OATS (Table 6). Cell viability (Section 4.3.1) and proliferation rate (Section 4.3.4) were assessed for each treatment after 24 h of cryopreservation. Following statistical analysis of viability across all treatment groups, the optimal preculture and cryoprotectant protocol was determined. A comparative analysis of proliferation rates across all treatment groups was performed, which validated the superior recovery performance of the optimal treatment group. EC without preculture and cryoprotectant treatment served as the directly cryopreserved control, with three replicates per treatment.

#### 4.3.3. Thawing Treatment

After 24 h of cryopreservation, the cryovials were retrieved and thawed at 4 °C (refrigerator), 25 °C (ambient temperature), 37 °C, 42 °C, or 50 °C (water bath). Following complete EC thawing, the cryoprotectant solution was removed. The EC within each cryovial was washed 3 to 4 times with liquid proliferation medium. EC was then placed on sterile filter paper to absorb excess liquid before being transferred onto solid proliferation medium for subsequent cell viability assessment. Based on the cell viability (Section 4.3.1) results, the optimal thawing temperature was selected.

#### 4.3.4. Proliferation Rate

The proliferation rate serves as a functional metric reflecting not only the survival of EC post-thaw but also the recovery capacity of the surviving cells. One day post-thawing, 0.1 g EC was randomly selected from the solid proliferation medium and transferred to fresh medium to thoroughly eliminate residual DMSO and PEG solutions. Following a 4-week recovery period, the samples were weighed under aseptic conditions using an analytical balance with a precision of 0.01 g to calculate the proliferation rate, with three replicates per treatment.

#### 4.3.5. Effect of Cryopreservation Duration (Storage Duration in Liquid Nitrogen) on Cell Viability and Proliferation Rate

EC subjected to preculture and cryoprotectant treatment were preserved in liquid nitrogen for 1 day, 7 days, 3 months, 6 months, or 1 year. After each cryopreservation period, post-thaw cell viability (Section 4.3.1) and post-recovery proliferation rate (Section 4.3.4) were assessed to analyze the effect of duration on cryopreservation efficacy. Three independent replicates were established for each cryopreservation duration.

#### 4.3.6. Assessment of Recovery Rate

The recovery rate serves as a reliable indicator of protocol stability. Ten cryovials cryopreserved for 4 months were retrieved and thawed. Under aseptic conditions, the *L. olgensis* EC from each cryovial was individually weighed using an analytical balance with a precision of 0.01 g. The EC was then transferred to solid proliferation medium and cultured in the dark at 25 °C. After 4 weeks, the EC was weighed again to assess recovery status. The recovery rate was calculated based on the number of recovery clumps.

#### 4.3.7. Validation of Somatic Embryogenic Potential

Cryopreserved EC was thawed and subjected to proliferation according to the methods described in previous sections. Following at least one subculture cycle (15 days), 0.2 g EC was transferred onto pre-maturation medium. Cultures were maintained at 25 °C under dark conditions for 10 days. Subsequently, the EC was transferred to somatic embryo maturation medium. After approximately 40 days of culture on somatic embryo maturation medium, somatic embryos were carefully separated from the EC and transferred onto germination medium. Germination typically occurred within about 5 days. Following germination, root development was observed after approximately 20 days, leading to the regeneration of complete plantlets.

### 4.4. Statistical Analysis

The proliferation rate, cell viability, and recovery rate of cryopreserved EC were calculated using the following formulas:

(1) $$\mathrm{Proliferation}\;\mathrm{rate}\;(\%)\;=\;\frac{\mathrm{Fresh}\;\mathrm{weight}\;\mathrm{of}\;\mathrm{regenerated}\;\mathrm{EC}-0.1\;(g)}{0.1\;(g)}\;\times \;100$$

(2) $$\mathrm{Cell}\;\mathrm{viability}\;(\%)=\;\frac{\mathrm{Absorbance}\;\mathrm{of}\;\mathrm{cryopreserved}\;\mathrm{EC}}{\mathrm{Absorbance}\;\mathrm{of}\;\mathrm{non-cryopreserved}\;\mathrm{EC}}\;\times \;100$$

(3) $$\mathrm{Recovery}\;\mathrm{rate}\;(\%)=\frac{\mathrm{Number}\;\mathrm{of}\;\mathrm{recovered}\;\mathrm{EC}\;\mathrm{clumps}}{\mathrm{Total}\;\mathrm{number}\;\mathrm{of}\;\mathrm{cryopreserved}\;\mathrm{EC}\;\mathrm{clumps}}\;\times \;100$$

The analysis of variance (ANOVA) was performed using SPSS Statistics 23.0. The OATS configuration was employed to identify the optimal combination of preculture and cryoprotectant conditions. The multiple comparisons were analyzed via the Least Significant Difference (LSD) program in SPSS Statistics 23.0. Values are presented as the mean ± SD.

## Figures and Tables

**Table 1:** Analysis of cell viability after preculture and cryoprotectant treatments using OATS L_16_ (4^4^).

Treatments	Factors				Cell Viability (%)
	Preculture Duration (T)	Preculture Strategy (M)	DMSO (D)	PEG (P)	
1	T_1_	**M_1_**	**D_1_**	P_1_	3.889
2	T_3_	M_3_	D_1_	**P_3_**	4.500
3	T_4_	M_4_	D_1_	P_4_	1.778
4	**T_2_**	M_2_	D_1_	P_2_	4.111
5	T_2_	M_4_	D_3_	P_1_	2.667
6	T_4_	M_3_	D_2_	P_1_	2.611
7	T_3_	M_2_	D_4_	P_1_	2.722
8	T_1_	M_4_	D_4_	P_3_	0.889
9	T_4_	M_1_	D_4_	P_2_	2.778
10	T_1_	M_3_	D_3_	P_2_	0.389
11	T_2_	M_3_	D_4_	P_4_	2.333
12	T_2_	M_1_	D_2_	P_3_	4.833
13	T_3_	M_1_	D_3_	P_4_	4.000
14	T_3_	M_4_	D_2_	P_2_	2.444
15	T_4_	M_2_	D_3_	P_3_	2.222
16	T_1_	M_2_	D_2_	P_4_	2.500
K_1_	23.000	**46.500**	**42.833**	35.667	
K_2_	**41.833**	34.667	37.167	37.167	
K_3_	41.000	29.500	27.833	**37.333**	
K_4_	28.167	23.333	26.167	31.833	
`X_1_	1.917	**3.875**	**3.569**	2.972	
`X_2_	**3.486**	2.889	3.097	3.097	
`X_3_	3.417	2.458	2.319	**3.111**	
`X_4_	2.347	1.944	2.181	2.653	
R	1.569	1.931	1.389	0.458	

K_i_ denotes the sum of observed values at the same factor level; `X_i_ represents the mean of observed values across different levels of a factor; R indicates the range. Specifically: T_1_ = 12 h, T_2_ = 24 h, T_3_ = 36 h, T_4_ = 48 h; M_1_ = 0.2 mol∙L^−1^ + 0.4 mol∙L^−1^ sucrose, M_2_ = 0.2 mol∙L^−1^ + 0.4 mol∙L^−1^ sorbitol, M_3_ = 0.4 mol∙L^−1^ sucrose, M_4_ = 0.4 mol∙L^−1^ sorbitol; D_1_ = 2.5%, D_2_ = 5%, D_3_ = 10%, D_4_ = 15%; P_1_ = 0%, P_2_ = 5%, P_3_ = 10%, P_4_ = 15%. Bold numbers indicate the highest values, and bold letters are the best levels.

**Table 2:** Multiple comparisons of cell viability in *L. olgensis* EC across factor levels.

Levels	Preculture Duration (h)	Preculture Strategy	DMSO (%)	PEG (%)
1	1.917 ± 1.483 B	3.875 ± 0.951 A	3.569 ± 1.366 A	2.972 ± 0.813
2	3.486 ± 1.375 A	2.889 ± 0.941 B	3.097 ± 1.240 A	2.431 ± 1.508
3	3.417 ± 1.086 A	2.458 ± 1.719 BC	2.320 ± 1.433 B	3.111 ± 1.849
4	2.347 ± 0.694 B	1.945 ± 0.903 C	2.181 ± 0.941 B	2.653 ± 1.043

Preculture duration level: 1 = 12 h, 2 = 24 h, 3 = 36 h, 4 = 48 h; Preculture strategy level: 1 = 0.2 mol∙L^−1^ + 0.4 mol∙L^−1^ sucrose, 2 = 0.2 mol∙L^−1^ + 0.4 mol∙L^−1^ sorbitol, 3 = 0.4 mol∙L^−1^ sucrose, 4 = 0.4 mol∙L^−1^ sorbitol; DMSO level: 1 = 2.5%, 2 = 5%, 3 = 10%, 4 = 15%; PEG level: 1 = 0%, 2 = 5%, 3 = 10%, 4 = 15%. See also Table 1, where different uppercase letters indicate significant differences at the *p* < 0.01 level, absence of letters indicates no significant difference at the *p* > 0.05 level.

**Table 3:** Multiple comparisons of cell viability in *L. olgensis* EC across 16 treatments, control, and optimized protocol.

Treatments	Factors				Cell Viability (%)
	Preculture Duration (T)	Preculture Strategy (M)	DMSO (D)	PEG (P)	
1	T_1_	M_1_	D_1_	P_1_	3.889 ± 0.59 AB
2	T_3_	M_3_	D_3_	P_3_	4.500 ± 1.17 A
3	T_4_	M_4_	D_4_	P_4_	1.778 ± 0.67 CDE
4	T_2_	M_2_	D_2_	P_2_	4.111 ± 1.18 A
5	T_2_	M_2_	D_2_	P_2_	2.667 ± 0.76 BC
6	T_4_	M_4_	D_4_	P_4_	2.611 ± 1.00 BC
7	T_3_	M_3_	D_3_	P_3_	2.722 ± 0.10 BC
8	T_1_	M_1_	D_1_	P_1_	0.889 ± 0.48 EF
9	T_4_	M_4_	D_4_	P_4_	2.778 ± 0.19 BC
10	T_1_	M_1_	D_1_	P_1_	0.389 ± 0.25 F
11	T_2_	M_2_	D_2_	P_2_	2.333 ± 1.04 CD
12	T_2_	M_2_	D_2_	P_2_	4.833 ± 1.01 A
13	T_3_	M_3_	D_3_	P_3_	4.000 ± 0.6 A
14	T_3_	M_3_	D_3_	P_3_	2.444 ± 0.59 C
15	T_4_	M_4_	D_4_	P_4_	2.222 ± 0.51 CD
16	T_1_	M_1_	D_1_	P_1_	2.500 ± 0.17 C
control	-	-	-	-	1.111 ± 0.42 DEF
optimized protocol	T_2_	M_1_	D_1_	P_3_	5.111 ± 0.1 A

T_1_ = 12 h, T_2_ = 24 h, T_3_ = 36 h, T_4_ = 48 h; M_1_ = 0.2 mol∙L^−1^ + 0.4 mol∙L^−1^ sucrose, M_2_ = 0.2 mol∙L^−1^ + 0.4 mol∙L^−1^ sorbitol, M_3_ = 0.4 mol∙L^−1^ sucrose, M_4_ = 0.4 mol∙L^−1^ sorbitol; D_1_ = 2.5%, D_2_ = 5%, D_3_ = 10%, D_4_ = 15%; P_1_ = 0%, P_2_ = 5%, P_3_ = 10%, P_4_ = 15%; different uppercase letters indicate significant differences at the *p* < 0.01 level.

**Table 4:** Multiple comparisons of proliferation rate in *L. olgensis* EC across 16 treatments, control, and optimized protocol.

Treatments	Factors				Proliferation Rate (%)
	Preculture Duration (T)	Preculture Strategy (M)	DMSO (D)	PEG (P)	
1	T_1_	M_1_	D_1_	P_1_	0.00 ± 0.00 F
2	T_3_	M_3_	D_3_	P_3_	370.00 ± 27.00 C
3	T_4_	M_4_	D_4_	P_4_	0.00 ± 0.00 F
4	T_2_	M_2_	D_2_	P_2_	340.33 ± 4.04 C
5	T_2_	M_2_	D_2_	P_2_	30.00 ± 17.32
6	T_4_	M_4_	D_4_	P_4_	66.67 ± 32.15 E
7	T_3_	M_3_	D_3_	P_3_	0.00 ± 0.00 F
8	T_1_	M_1_	D_1_	P_1_	0.00 ± 0.00 F
9	T_4_	M_4_	D_4_	P_4_	0.00 ± 0.00 F
10	T_1_	M_1_	D_1_	P_1_	0.00 ± 0.00 F
11	T_2_	M_2_	D_2_	P_2_	0.00 ± 0.00 F
12	T_2_	M_2_	D_2_	P_2_	480.00 ± 30.00 B
13	T_3_	M_3_	D_3_	P_3_	276.67 ± 30.55 D
14	T_3_	M_3_	D_3_	P_3_	0.00 ± 0.00 F
15	T_4_	M_4_	D_4_	P_4_	0.00 ± 0.00 F
16	T_1_	M_1_	D_1_	P_1_	0.00 ± 0.00 F
control	-	-	-	-	0.00 ± 0.00 F
optimized protocol	T_2_	M_1_	D_1_	P_3_	713.33 ± 11.55 A

T_1_ = 12 h, T_2_ = 24 h, T_3_ = 36 h, T_4_ = 48 h; M_1_ = 0.2 mol∙L^−1^ + 0.4 mol∙L^−1^ sucrose, M_2_ = 0.2 mol∙L^−1^ + 0.4 mol∙L^−1^ sorbitol, M_3_ = 0.4 mol∙L^−1^ sucrose, M_4_ = 0.4 mol∙L^−1^ sorbitol; D_1_ = 2.5%, D_2_ = 5%, D_3_ = 10%, D_4_ = 15%; P_1_ = 0%, P_2_ = 5%, P_3_ = 10%, P_4_ = 15%; different uppercase letters indicate significant differences at the *p* < 0.01 level.

**Table 5:** Culture medium names and formulations.

Names	Formulations
Preculture medium	BM supplemented with 0.15 mg·L^−1^ 2,4-D, 0.05 mg·L^−1^ 6-BA, 0.05 mg·L^−1^ KT, 1 g·L^−1^ L-glutamine, 1 g·L^−1^ inositol, 0.5 g·L^−1^ casein acids hydrolysate, 5.2 g·L^−1^ agar and 0.2 mol·L^−1^/0.4 mol·L^−1^ sucrose/sorbitol, pH of 6.0
Liquid proliferation medium	BM supplemented with 0.15 mg·L^−1^ 2,4-D, 0.05 mg·L^−1^ 6-BA, 0.05 mg·L^−1^ KT, 1 g·L^−1^ L-glutamine, 1 g·L^−1^ inositol, 0.5 g·L^−1^ casein acids hydrolysate and 25 g·L^−1^ sucrose, pH of 6.0
Solid proliferation medium	BM supplemented with 0.15 mg·L^−1^ 2,4-D, 0.05 mg·L^−1^ 6-BA, 0.05 mg·L^−1^ KT, 1 g·L^−1^ L-glutamine, 1 g·L^−1^ inositol, 0.5 g·L^−1^ casein acids hydrolysate, 5.2 g·L^−1^ agar and 25 g·L^−1^ sucrose, pH of 6.0
Pre-maturation medium	1/4 BM supplemented with 1 g·L^−1^ L-glutamine, 10 g·L^−1^ inositol, 0.5 g·L^−1^ casein acids hydrolysate, 5.2 g·L^−1^ agar and 60 g·L^−1^ sucrose, pH of 6.0
Somatic embryo maturation medium	BM supplemented with 20 mg·L^−1^ abscisic acid, 80 g·L^−1^ PEG_6000_, 0.5 g·L^−1^ L-glutamine, 0.5 g·L^−1^ inositol, 0.25 g·L^−1^ casein acids hydrolysate, 2 g·L^−1^ Gelzan^TM^ CM and 60 g·L^−1^ sucrose, pH of 6.0
Germination medium	1/2 MS supplemented with sucrose 30 g·L^−1^ and agar 6.5 g·L^−1^, pH of 5.8

**Table 6:** Screening for optimal preculture and cryoprotectant conditions of *L. olgensis* EC using OATS.

Levels	Preculture Duration (T) (h)	Preculture Strategy (M)	DMSO (D) (%)	PEG (P) (%)
1	12	0.2 mol·L^−1^ + 0.4 mol·L^−1^ sucrose	2.5	0
2	24	0.2 mol·L^−1^ + 0.4 mol·L^−1^ sorbitol	5	5
3	36	0.4 mol·L^−1^ sucrose	10	10
4	48	0.4 mol·L^−1^ sorbitol	15	15

## Data Availability

All data are available in the manuscript.

## Abbreviations

The following abbreviations are used in this manuscript:

EC	Embryogenic callus
SE	Somatic embryogenesis
DMSO	Dimethyl Sulfoxide
PEG_6000_	Polyethylene Glycol 6000
TTC	2,3,5-triphenyltetrazolium chloride
OATS	Orthogonal array testing strategy
